# Repeated Three-Hour Maternal Separation Induces Depression-Like Behavior and Affects the Expression of Hippocampal Plasticity-Related Proteins in C57BL/6N Mice

**DOI:** 10.1155/2015/627837

**Published:** 2015-12-22

**Authors:** Yaoyao Bian, Lili Yang, Zhongli Wang, Qing Wang, Li Zeng, Guihua Xu

**Affiliations:** ^1^School of Nursing, Nanjing University of Chinese Medicine, Nanjing 210023, China; ^2^Medical College, Nantong University, Nantong 226001, China; ^3^School of Basic Medical Sciences, Nanjing University of Chinese Medicine, Nanjing 210023, China; ^4^First Clinical Medical College, Nanjing University of Chinese Medicine, Nanjing 210023, China

## Abstract

Adverse early life experiences can negatively affect behaviors later in life. Maternal separation (MS) has been extensively investigated in animal models in the adult phase of MS. The study aimed to explore the mechanism by which MS negatively affects C57BL/6N mice, especially the effects caused by MS in the early phase. Early life adversity especially can alter plasticity functions. To determine whether adverse early life experiences induce changes in plasticity in the brain hippocampus, we established an MS paradigm. In this research, the mice were treated with mild (15 min, MS15) or prolonged (180 min, MS180) maternal separation from postnatal day 2 to postnatal day 21. The mice underwent a forced swimming test, a tail suspension test, and an open field test, respectively. Afterward, the mice were sacrificed on postnatal day 31 to determine the effects of MS on early life stages. Results implied that MS induces depression-like behavior and the effects may be mediated partly by interfering with the hippocampal GSK-3*β*-CREB signaling pathway and by reducing the levels of some plasticity-related proteins.

## 1. Introduction

In mammals, adverse life events that occur in early neuronal development can change normal brain growth and stress vulnerability in adulthood [1]. Acute or chronic stressful periods, particularly during childhood and adolescence, induce the onset of emotional and affective disorders, such as depression and anxiety [2]. The time and duration of any stressful experience that occurs in the neonatal or adolescent period are possibly necessary to promote proper neuronal organization; these parameters can also exacerbate the vulnerability to long-term behavioral changes [3]. Our study generally aimed to assess the mechanism by which the association between neonatal and adolescent stressful experiences may influence stress responsiveness and brain plasticity in C57BL/6N mice.

We induced MS in male mice, an established model of adverse early life experiences. Considering depression, researchers hypothesized that structural plasticity and neurotrophic factors are necessary to mediate behavioral responses to MS. For example, neurofilament light chain (NF-L) is a reliable marker of structural plasticity; this marker indicates neuronal impairment at a molecular level. NF-L is also a subunit of neurofilaments (NFs). NFs are neuron-specific cytoskeletal filaments found in most mature neurons. NFs provide structural support for neurons and their synapses; NFs also maintain and control neuronal cytoskeletal plasticity by regulating neurite outgrowth, axonal caliber, and axonal transport [4]. In addition to NF-L, brain-derived neurotrophic factor (BDNF) is a key regulator of neuronal plasticity. BDNF strongly affects synaptogenesis, spine formation [5], neuronal survival [6], long-term potentiation, neuronal excitability [7], and adult hippocampal neurogenesis [8]. The transcription of several genes, such as BDNF, is stimulated by activating the phosphorylation of cAMP response element-binding protein (CREB) (Ser133) [9]. CREB is regarded as a key nucleoprotein related to depression and antidepressant treatments [10].

The transduction pathways and upstream signaling molecules of CREB-BDNF are complex; among these molecules, glycogen synthase kinase-3 (GSK-3) is a newly reported inhibitory signaling molecule [11–13]. This kinase was originally identified as a key enzyme of glucose metabolism. GSK-3 is considered as a broadly influential enzyme that affects a diverse range of biological functions because this enzyme regulates a large group of transcription factors and transcriptional modulators [14]. GSK-3 can be directly inhibited by the mood stabilizer lithium [15]; this result suggests that GSK-3 may be associated with the pathophysiology of mood disorders. Furthermore, GSK-3 exists in two closely related isoforms, namely, GSK-3*α* and GSK-3*β*. The constitutively active GSK-3*β* is an important regulatory protein involved in many neuroplasticity-associated intracellular signaling pathways [16]. In our study, the MS-induced depression-like behavior and some hippocampal plasticity-related proteins were observed in male C57BL/6N mice; the effects on the MS model were then investigated. The underlying mechanism was also determined on the basis of the GSK-3*β*-CREB signaling pathway.

## 2. Materials and Methods

### 2.1. Experimental Animals

The pregnant C57BL/6N mice were obtained from the Laboratory Animal Center of Nanjing University of Chinese Medical. The mice were housed in groups of four in home cages made of Plexiglas (35 cm × 15 cm × 10 cm) with sawdust bedding. The animals were maintained under a standard dark-light cycle (lights on between 6:00 and 18:00) at room temperature of 22 ± 2°C. The mice had free access to food and water. Prior to the experiments, mice were habituated to daily handling during the week after delivery. All animals treatments were in accordance with the Guidelines of Accommodation and Care of Animals formulated by the Chinese Convention for the Protection of Vertebrate Animals used for experimental and other scientific purposes. All efforts were made to minimize animal suffering and reduce the number of animals used for experiments.

### 2.2. Experimental Design

**Figure 1:**
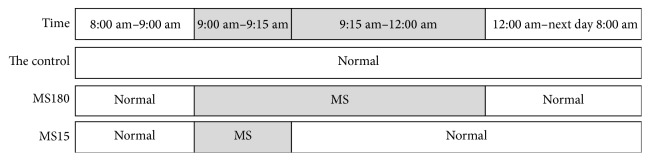
MS180 consisted of daily separation of litters from their dams and sires for 3 h (09:00–12:00 h), while MS15 involved daily brief separations of 15 min (09:00–09:15 h).

The mouse was born at the first day postpartum (PD1), respectively, from PD2 to PD21 of MS (MS took place when the mouse was moved to a single cage); the mice were moved to the incubator (30 to 32°C). MS180 consisted of daily separation of litters from their dams and sires for 3 h (09:00–12:00 h), while MS15 involved daily brief separations of 15 min (09:00–09:15 h), as shown in Figure 1.

### 2.3. Behavioral Tests

Behavioral tests were performed in JLBehv-FSG-4 sound insulation boxes with the DigBehav animal behavior video analysis system (Shanghai Jiliang Software Technology Co., Ltd., Shanghai, China). DigBehav can automatically record and analyze animal movements to provide total immobility times during the FST and TST. Depression-like behavior was inferred from the increasing time spent immobile during these tests. The FST method was similar to that described by Porsolt et al. [17]. Considering the younger ages of our experimental mice, shorter body length, and the optimization of our preexperiment, the mice were placed individually in 10 cm deep water at ambient temperature (25 ± 1°C) in a 2000 mL glass beakers and were allowed to swim for 5 minutes. Plus, the strength of our mice was weaker compared with adult ones. Taking the above into account, the adaptation time was shortened to 1 minute. The duration of immobility was recorded during the last 4 min of the test. The TST method was similar to that described by Steru et al. [18]. After the FST, the mice were allowed to rest for 24 h. Each mouse was then suspended on the edge of a shelf at 58 cm above the bottom of the sound insulation box, using adhesive tape placed approximately 1 cm from the tip of the tail. The animals were allowed to hang for 6 min, and the duration of immobility was recorded during the last 4 min of the test.

### 2.4. Real-Time PCR Analysis

Total RNA from bilateral hippocampal tissue was extracted using Trizol reagent (cwbio, cw0580). cDNA was synthesized with 2 *μ*g of total RNA using the RevertAid Transcript First-Strand cDNA Synthesis Kit (Fermentas, K1622). Quantitative real-time PCR was performed using the SYBR Green Master Mix (Fermentas, K0222) in the StepOne Real-Time PCR System (ABI, USA). The sequences of primers were BDNF forward: 5′-GGTCACAGCGGCAGATAAAAAGAC-3′, reverse: 5′-TTGGGTAGTTCGGCATTGCGAG-3′; NF-L forward: 5′-GTTCAAGAGCCGCTTCACCG-3′, reverse: 5′-CCAGGGTCTTAGCCTTGAGCAG-3′; GAPDH forward: 5′-TGAAGGTCGGAGTCAACGGATTTGGT-3′, reverse: 5′-CATGTGGGCCATGAGGTCCACCAC-3′. The following thermal cycling conditions were used: initial denaturation at 95°C for 10 min, followed by 40 cycles of denaturation at 95°C for 15 s and then annealing and extension, both at 60°C for 1 min. The amplification of only a single sequence was verified by the dissociation curve of each reaction. All experiments were performed in triplicate, and the average threshold cycle (Ct) value was the extreme Ct value of the sample. The mRNA expression of GFAP was calculated relative to the house keeping gene GFAP using the 2^−ΔCt^ method, ΔCt = Ct_(the  target  gene)_ − Ct_(GFAP)_.

### 2.5. Western Blot Analysis

Bilateral hippocampal tissue samples were homogenized at 4°C in 0.5 mL of lysis buffer containing 50 mM Tris-HCl, 0.1% sodium dodecyl sulfate (SDS), 1% Nonidet-P40 (NP-40, Sigma), 1 mM EDTA, 150 mM NaCl, 1 mM phenylmethylsulfonyl fluoride (Sigma), 1 mM NaF, 1 mM Na_3_VO_4_, 1 *μ*gmL^−1^ aprotinin (Sigma), and 1 *μ*gmL^−1^ leupeptin (Sigma) (pH 7.5). Aliquots of the clarified homogenized liquid, containing 75 *μ*g of protein, were denatured at 95°C for 5 min in a sample buffer containing 1% SDS, 1% dithiothreitol (Sigma), 10 mM Tris-HCl, 10% glycerol, and 1 mM EDTA (pH 8.0). The sample proteins were then separated by 12% SDS-polyacrylamide gel electrophoresis and transferred to polyvinylidene fluoride membranes (Bio-Rad). The primary antibodies used to examine the changes in protein expression included the rabbit polyclonal anti-BDNF antibody (1 : 200, Abcam, ab6201), the mouse monoclonal anti-NF-L antibody (1 : 500, Invitrogen, 13-0400), the rabbit monoclonal anti-CREB (1 : 1000, Cell Signaling, 9197S), the rabbit monoclonal phospho-CREB (Ser133) (1 : 1000, Cell Signaling, 9198S), the rabbit monoclonal anti-GSK-3*β* (1 : 1000, Cell Signaling, 9315S), the rabbit monoclonal anti-phospho-GSK-3*β* (Ser9) (1 : 1000, Cell Signaling, 9323S), and the mouse monoclonal anti-*β*-actin (1 : 2000, Sigma, A1978). The secondary antibodies included the horseradish peroxidase conjugated goat anti-mouse IgG (1 : 4000, GenScript) and the goat anti-rabbit IgG (1 : 4000, GenScript). Immunoblotting was detected by enhanced chemiluminescence (Bio-Rad XRS+) and analyzed using ImageLab 5.0. The values of the BDNF, NF-L, CREB, and GSK-3*β* levels were normalized against the amount of *β*-actin obtained from the same sample. The phospho-GSK-3*β*/GSK-3*β* and phospho-CREB/CREB were calculated to reflect the activity of GSK-3*β* and CREB. Three protein samples per animal were examined for each target protein.

### 2.6. Statistical Analysis

Data were expressed as the mean ± SEM for the indicated number of experiments and analyzed using the Statistical Package for Social Sciences computer program (version 20.0). The statistical significance of the results was determined using one-way ANOVA, followed by Tukey's post hoc tests. The significance level was set at *P* ≤ 0.05 for all statistical comparisons.

## 3. Results

### 3.1. Effects of Maternal Separation on Body Weight

Offspring weight was assessed on PND2, PND7, PND14, PND21, and PND28. A difference in weight was observed on PND14 (*F*_2,27_ = 4.07, *P* < 0.05), PND21 (*F*_2,27_ = 5.74, *P* < 0.01), and PND28 (*F*_2,27_ = 6.11, *P* < 0.01), where in MS180, the body weight were lighter than Control and MS15 since PND14 to PND28 (Table 1).

### 3.2. Effects of Maternal Separation on Immobility Time in the Mouse TST

**Figure 2:**
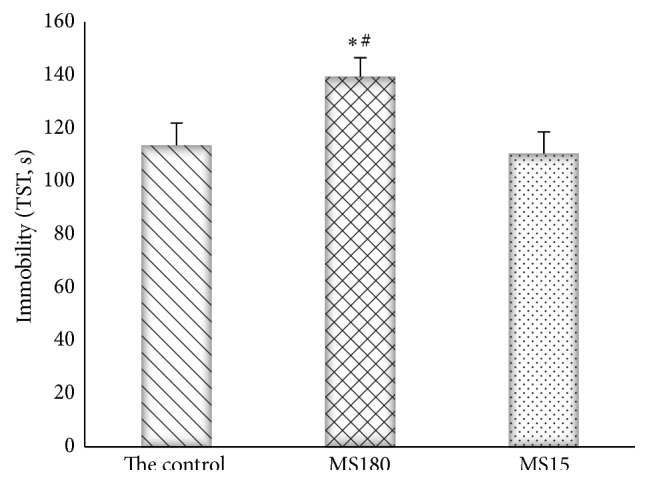
The tail suspension immobility time in animal testing. ^*∗*^ *P* < 0.01 versus the control group, ^#^ *P* < 0.01 versus the MS15 group.

In TST (*F*_2,27_ = 3.69, *P* < 0.05), the immobility times differed significantly among the groups. Multiple comparison tests revealed that MS180 induced a significant increase in immobility time compared with the control group and the MS15 group in the TST (*P* < 0.01). MS15 and the control group had no significant difference (*P* > 0.05) (Figure 2).

### 3.3. Effects of Maternal Separation on Immobility Time in the Mouse FST

**Figure 3:**
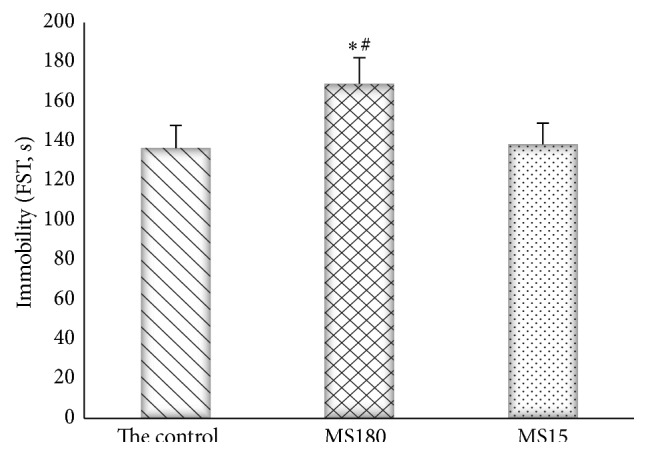
The forced swimming test of static time. ^*∗*^ *P* < 0.01 versus the control group, ^#^ *P* < 0.01 versus the MS15 group.

In FST (*F*_2,27_ = 4.52, *P* < 0.05), the immobility times differed significantly among the groups. Multiple comparison tests revealed that MS180 induced a significant increase in immobility time compared with the normal group and MS15 in the FST (*P* < 0.01). MS15 and the control group had no significant difference (*P* > 0.05) (Figure 3).

### 3.4. Effects of Maternal Separation on Distance in the Mouse Open Field Test

**Figure 4:**
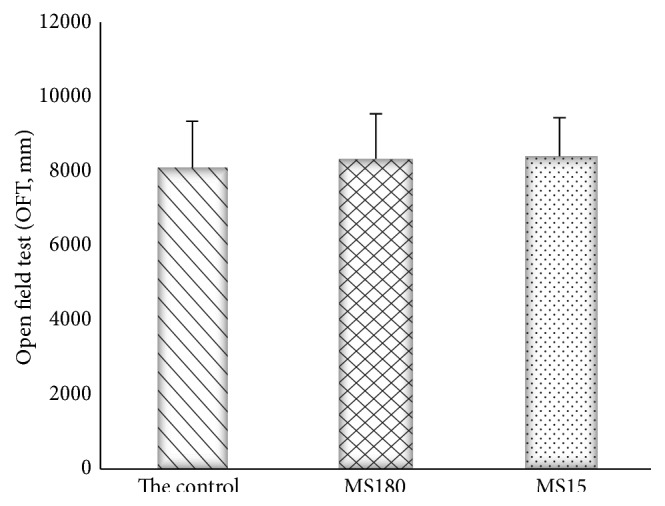
The open field test on shifting distance. *P* > 0.05 versus the control group and the MS15 group.

In open field test (*F*_2,27_ = 0.409, *P* > 0.05), the results showed no statistical significance between groups (Figure 4).

### 3.5. Maternal Separation Reduced the Hippocampal mRNA Levels of BDNF and NF-L in C57BL/6N Mice

**Figure 5:**
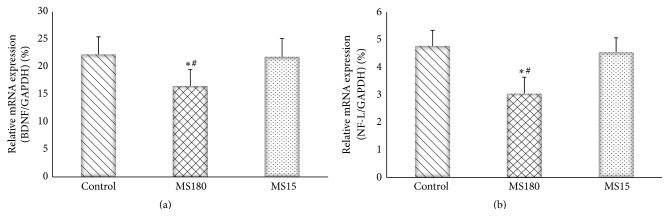
Effects of MS on the hippocampal mRNA levels of BDNF (a) and NF-L (b) in the mouse model. ^*∗*^ *P* < 0.01 versus the control group and ^#^ *P* < 0.01 versus the MS15 group.

The relative target gene mRNA levels of the groups are shown in Figure 5. The ANOVA tests showed a significant effect of the groups in the hippocampal mRNA level of BDNF (*F*_2,27_ = 7.94, *P* < 0.01) and NF-L (*F*_2,27_ = 8.29, *P* < 0.01). Post hoc comparisons revealed that the MS180 significantly decreased the hippocampal mRNA levels of BDNF and NF-L compared with the control group and MS15 (*P* < 0.01).

### 3.6. Maternal Separation Reduced the Hippocampal Protein Levels of GSK-3*β* Inhibitory Phosphorylation, CREB Activation, BDNF, and NF-L

**Figure 6:**
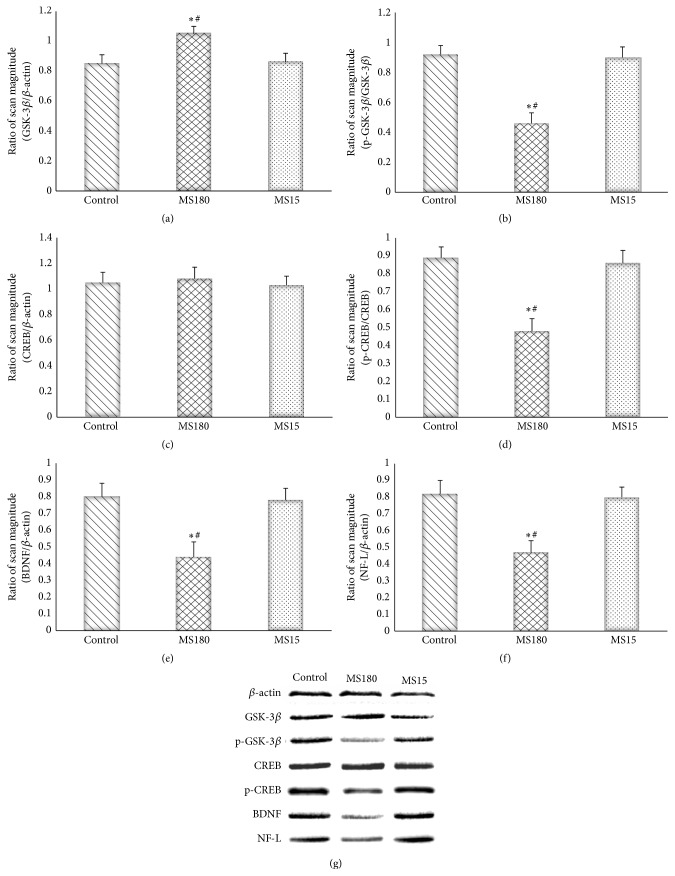
Hippocampal GSK-3*β*, p-GSK-3*β*, CREB, p-CREB, BDNF, and NF-L protein levels in the MS-induced mouse model were determined by Western blot analysis. The values of GSK-3*β* (a), CREB (c), BDNF (e), and NF-L (f) levels were normalized against the amount of *β*-actin, while the values of p-GSK-3*β* (Ser9) (b) and p-CREB (Ser133) (d) were normalized against the amount of GSK-3*β* and CREB, respectively. ^*∗*^ *P* < 0.01 versus the control group, ^#^ *P* < 0.01 versus the MS15 group.

Figure 6 shows that the hippocampal protein level of GSK-3*β* was significantly higher in the MS180 group than in the control group and the MS15 group (*F*_2,27_ = 5.77, *P* < 0.01). The ratios of phospho-GSK-3*β* (Ser9) and GSK-3*β* were significantly different among the groups (*F*_2,27_ = 7.13, *P* < 0.01). By contrast, the expression of the hippocampal CREB protein was not significantly different among the groups (*F*_2,27_ = 1.46, *P* > 0.05). Post hoc comparisons revealed that the ratio of phospho-CREB (Ser133) was lower in the MS180 group than in the control group and the MS15 group (*F*_2,27_ = 11.07, *P* < 0.01). The hippocampal protein levels of BDNF and NF-L in the MS180 group were significantly lower than those in the control group and the MS15 group (*F*_2,27_ = 8.53, *P* < 0.01).

## 4. Discussion

Previous studies revealed that MS induced acutely or subacutely to normal mice elicits depression-like effects [19–22]. However, previous studies focused on the changes in the adult phase of MS. Few studies have demonstrated the effects of MS on the changes in the early phase of mice, although the early life stages of mice are a key period in the development of the nervous system. Hence, further studies should be conducted to explore the effects of MS on early life stages.

Our study demonstrated that MS180 significantly reduced the immobility time in FST and/or TST. Depression affects spontaneous locomotor activity [23]; as such, the reduced immobility time may account for the MS-induced depression. The behavioral results of our study are consistent with those described in previous studies, which examined the effects of MS. However, the mice in our study were monitored on postnatal day 31, not on the usual age of more than eight weeks.

Consistent with previous findings, our results confirmed that MS180 induced depression-like behavior and hippocampal impairment in mice. These results were based on the increased immobility time in the behavioral tests and the reduced expression of BDNF and NF-L in the hippocampus. Moreover, MS180 evidently induced these depression-like effects; by contrast, MS15 did not significantly affect the BDNF and NF-L protein levels and the behavioral test results. Considering that MS15 did not significantly reduce the BDNF or NF-L expression in the hippocampus of normal mice, we concluded that MS induced the depression-like behavior as a result of the decreased expression of these plasticity-related proteins; thus, neuroplasticity may be inhibited.

Neuroplasticity-related signaling pathways may be involved in the pathophysiology and mechanisms of depression [24, 25]. In our study, this issue was addressed by investigating the involvement of the CREB-BDNF signaling pathway in the hippocampus. The downregulation of the hippocampal BDNF expression has been demonstrated in various animal depression models and in depressed patients; the chronic treatment of several classes of antidepressants increases the BDNF expression [26]. As an upstream transcriptional activator of BDNF, the hippocampal CREB expression is decreased among experimental animals exposed to specific stressors [27, 28]. A decrease in the CREB expression has also been observed in depressed patients [29, 30]. Our results showed that MS180 normalized the downregulated hippocampal mRNA and protein levels of BDNF; MS180 also reduced the activation of CREB in the C57BL/6N mouse model. This result further confirmed the depression-like effects of long-term MS; this result also suggested that the depression-like effects of MS180 may be due to the inhibition of CREB-BDNF in the hippocampus. Furthermore, MS15 did not induce any significant depression activity in the behavioral tests. MS15 could not also affect the mRNA expression of BDNF and the protein expression of phospho-CREB (Ser133) in the hippocampus. Therefore, short-term MS did not induce depression; by contrast, long-term MS could cause depression.

Previous studies on the effects of adverse early life experiences on the CNS focused on BDNF because of the unique role of this molecule in the CNS. However, studies have rarely investigated the mechanism by which MS influences the upstream signaling pathway of BDNF. Our study focused on GSK-3*β*, another upstream signaling molecule of CREB-BDNF, because GSK-3*β* is involved in various signaling systems [14] and is possibly associated with mood disorders [11].

GSK-3*β* and phospho-GSK-3*β* (Ser9) were also investigated in this study. Our results showed that MS180 increased the GSK-3*β* expression and reduced its inhibitory phosphorylation. Consistent with previous findings on depressed rats and patients [31, 32], our results further confirmed that insufficient GSK-3*β* inhibition is a risk factor of depression. In our study, MS180 significantly downregulated the inhibitory phosphorylation of GSK-3*β* in our model; thus, MS180 may activate GSK-3*β*. To the best of our knowledge, this study is the first to investigate the effect of MS on the early life phase of mice and to examine the hippocampal GSK-3*β* level and activity in this mouse model. This study is also the first to reveal the effects of MS on GSK-3*β* in this model. GSK-3*β* participates in several intracellular signaling pathways involved in neuroprotection [16]. Our results suggested that the GSK-3*β*-CREB signaling pathway may contribute to the decreased expression of some plasticity-related proteins in the hippocampus; this pathway may also induce depression-like behaviors. Moreover, the MS-induced activity of the GSK-3*β*-CREB signaling pathway is a possible mechanism of depression.

## 5. Conclusion

The structural plasticity of the hippocampus is critical for adverse early life experiences. In the MS model, MS180 induced depression-like behaviors and decreased the expression of some plasticity-related proteins. MS180 also inhibited the CREB-BDNF signaling pathway in the hippocampus. Furthermore, MS180 decreased the inhibitory phosphorylation of GSK-3*β*; as a result, the CREB-BDNF signaling pathway was inhibited. Thus, this inhibition may account for the depression-like activity of MS180.

## Figures and Tables

**Table 1:** Offspring weight across development.

Group	PND2	PND7	PND14	PND21	PND28
Control	1.58 ± 0.02	4.67 ± 0.07	7.14 ± 0.11	9.79 ± 0.23	12.34 ± 0.31
MS180	1.59 ± 0.02	4.62 ± 0.08	6.76 ± 0.14^*∗*^	9.34 ± 0.21^*∗∗*^	11.77 ± 0.28^*∗∗*^
MS15	1.58 ± 0.03	4.65 ± 0.06	7.11 ± 0.12	9.82 ± 0.20	12.32 ± 0.27

MS180 reduced body weight versus with control. ^*∗*^ *P* < 0.05 statistical significance to control and MS15. ^*∗∗*^ *P* < 0.01 significant difference to control and MS15. PND = postnatal day.
